# Contribution of Toll-Like Receptor 2 to the Innate Response against *Staphylococcus aureus* Infection in Mice

**DOI:** 10.1371/journal.pone.0074287

**Published:** 2013-09-13

**Authors:** Yimin, Masashi Kohanawa, Songji Zhao, Michitaka Ozaki, Sanae Haga, Guangxian Nan, Yuji Kuge, Nagara Tamaki

**Affiliations:** 1 Department of Advanced Medicine, Graduate School of Medicine, Hokkaido University, Kita-ku, Sapporo, Japan; 2 Department of Tracer Kinetics and Bioanalysis, Graduate School of Medicine, Hokkaido University, Kita-ku, Sapporo, Japan; 3 Faculty of Health Sciences, Graduate School of Medicine, Hokkaido University, Kita-ku, Sapporo, Japan; 4 Department of Neurology, China-Japan Union Hospital of Jilin University, Changchun, China; 5 Central Institute of Isotope Science, Hokkaido University, Kita-ku, Sapporo, Japan; 6 Department of Nuclear Medicine, Graduate School of Medicine, Hokkaido University, Kita-ku, Sapporo, Japan; Louisiana State University, United States of America

## Abstract

*Staphylococcus aureus* is a common pathogen that causes a wide range of infectious diseases. The function of TLRs, specifically TLR2, during *S. aureus* infection is still debated. In this study, we investigated the extent to which TLR2 contributes to the host innate response against the bacterial infection using TLR2-deficient mice. Intravenous inoculation with *S. aureus* resulted in all TLR2-deficient mice dying within 4 d, along with a high bacterial burden in the livers. However, histological examination showed the same degree of macrophage and neutrophil accumulation in the livers of infected TLR2-deficient mice as that in infected wild-type (WT) mice. TLR2-deficient mouse macrophages also showed normal phagocytic activity, although they failed to express CD36 that appeared on the surface of WT mouse cells upon challenge with heat-killed *S. aureus*. These data indicate that TLR2, as well as CD36, does not directly affect *S. aureus* clearance and that CD36 expression on macrophages depends on the presence of TLR2. *In vivo* infection with *S. aureus* caused significantly elevated production of TNF-α and IL-6 in the livers and blood of TLR2-deficient mice compared with those in WT mice, while the hepatic and serum levels of IL-10 decreased in these mice. In contrast, lower expression of IL-6 and IL-10, but not of TNF-α, at both the gene and protein levels was found in TLR2-deficient mouse macrophages compared to that in WT mouse cells, in response to challenge with heat-killed *S. aureus*. These findings suggest that the *S. aureus*-induced pro-inflammatory cytokine response is not dependent on macrophages and that TLR2 deficiency results in decreased IL-10 release by macrophages, which contributes to dysregulated cytokine balance, impaired bacterial clearance, and mouse death. Therefore, TLR2 possesses a protective function during *S. aureus* infection by regulating pro- and anti-inflammatory cytokine responses.

## Introduction

The gram-positive bacterium *Staphylococcus aureus* often asymptomatically colonizes the host’s nostrils, upper respiratory tract, and skin. *S. aureus* is also a major pathogen that causes a variety of diseases ranging from minor skin infections to severe systemic complications such as bacteremia and septic shock, often with fatal results [1]. *S. aureus* possesses a number of potential virulence factors including cell wall-associated and secreted proteins, which especially cause immunogenic and toxic injuries [2,3]. Several staphylococcal cell wall components (e.g., peptidoglycan and lipoteichoic acid) have been extensively studied to date [4,5,6,7]. However, bacterial pathogenicity in the case of *S. aureus* is multifactorial since a single virulence factor is not sufficient to cause a staphylococcal infection [8,9]. Therefore, it is important to understand the host response to exposure to the whole *S. aureus*.

Protection from primary staphylococcal infection is mainly dependent on innate rather than adaptive immune responses [10]. In the innate immune response, TLRs, which are predominantly expressed in cells involved in immuno-inflammatory responses, play pivotal roles in the host defense against microbial pathogens by recognizing pathogen-associated molecular patterns (PAMPs) and activating intracellular signaling pathways [11]. Among the known TLRs, TLR2 is a key sensor for detecting *S. aureus* invasion. There is increasing evidence that TLR2 found on monocytes/macrophages is involved in the detection of *S. aureus* PAMPs via TLR1 or TLR6 heterodimers and acts with other coreceptors to mediate phagocytosis of the bacterium [9,12]. Furthermore, TLR2 induces the synthesis of pro-inflammatory mediators to rapidly activate the innate immune system [13,14]. Recent evidence further suggests that TLR2 mediates the production of pro-inflammatory cytokines by non-immune cell populations, such as endothelial cells and hepatic stellate cells, in response to challenge with *S. aureus* or its PAMPs [15,16]. Interestingly, TLR2 deficit enhances the host’s susceptibility to staphylococcal infection, attenuates pro-inflammatory cytokine production, and results in high mortality [12,17,18]. Conversely, a recent report has shown that TLR2-deficient mice infected with *S. aureus* have elevated TNF-α and IL-6 levels that contribute to decreased survival in mice [7]. In addition, robust intradermal growth of *S. aureus* has been observed in TLR2-deficient mice after subcutaneous injection [12]. In contrast, the absence of TLR2 has been shown to provide protective effects to the host organism in certain models. For example, TLR2 deficiency attenuates the production of *S. aureus*-induced cardiac pro-inflammatory mediators and inhibits the development of myocardial dysfunction [19]. Thus, TLR2 has not only beneficial but also detrimental roles in host innate response to staphylococcal infections. However, the molecular mechanisms for these divergent roles, particularly those behind its effects *in vivo*, remain unclear. In addition to its role in immunity, TLR2 also induces *in vitro* compensatory responses to *S. aureus* infection that regulate epithelia homeostasis in an autonomous, non-inflammatory manner [20].

In the present study, we investigated the role of TLR2 in bacterial clearance and cytokine response to whole *S. aureus* infection in mice. Intravenous inoculation with *S. aureus* resulted in the death of all TLR2-deficient mice within 4 d with a high bacterial burden in their organs. The data presented here indicate that TLR2, as well as its coreceptor CD36, does not play a direct role in *S. aureus* clearance, but that TLR2 deficiency attenuates the macrophage release of IL-10, thereby leading to excessive pro-inflammatory cytokine response, bacterial proliferation, and mouse death during systemic *S. aureus* infection. This work may help us to better understand the host innate immune mechanism against this bacterium and will aid in the development of therapeutics for treating *S. aureus*. 

## Materials and Methods

### Mice

C57BL/6 TLR2-deficient mice and wild-type (WT) controls were purchased from Oriental Bioservice Inc. (Kyoto, Japan) and Charles River Laboratories Japan (Yokohama, Japan), respectively. After 7 d of acclimation, 8-wk-old female mice were used for *in vivo* infection studies, and the males were used for *in vitro* experiments. All the mice were housed in a temperature-controlled room with a 12-h light/dark diurnal cycle and had free access to food and drink throughout the experiment.

### Ethics statement

All experimental procedures were approved by the Institutional Animal Care and Use Committee of Hokkaido University (approval number 08-0073) and were performed under the control of the Guidelines for Animal Experiments at the Graduate School of Medicine, Hokkaido University.

### Experimental infections with *S. aureus*

Female mice were inoculated via a lateral tail vein with 3 × 10^7^ CFU of the viable *S. aureus* strain 834 suspended in 0.2 ml of PBS. Those injected with 0.2 ml of PBS alone served as controls. Mouse survival was monitored daily for up to 14 d post-infection, and survival curves were plotted.

### Bacterial counts in organs

Livers and kidneys were harvested from infected mice at the indicated times post-infection and homogenized in RPMI 1640 medium (Sigma, Mo., USA) (0.1 g/10 ml); 100 µl of the organ homogenates and their serial 10-fold dilutions were plated on nutrient agar plates. Viable colonies were counted at 24 h after culture. Using this method, we could detect >10^3^ bacteria/g of organ tissue.

### Sample preparation for cytokine assay

The livers and kidneys were aseptically removed from mice and suspended in RPMI 1640 medium containing 1% (w/v) CHAPS (Wako Pure Chemical Co., Kyoto, Japan). Ten-percent (w/v) homogenates were prepared with a Dounce grinder. The homogenates were cleared by centrifugation at 2,000 × *g* for 30 min at 4 °C, and the supernatants were stored at -80 °C until use in the cytokine assay. Blood samples were also collected by venipuncture into 1-ml sterile syringes containing heparin and were separated by centrifugation at 1,200 × *g* for 20 min at 4 °C. Separated plasma samples were aliquoted and stored at -80 °C for subsequent tests.

### Isolation and stimulation of peritoneal macrophages

Peritoneal macrophages were harvested from uninfected mice and suspended in 0.83% ammonium chloride solution containing 10% (v/v) Tris buffer (pH 7.65) to lyse erythrocytes. The cells were resuspended in RPMI 1640 medium supplemented with 10% FBS, 100 IU/ml penicillin, and 100 mg/ml streptomycin, and then plated in 24-well plates at 5 × 10^5^ cells/well for TNF-α and IL-6 ELISA assays and in 35-mm dishes at 2 × 10^6^ cells/dish for IL-10 ELISA assay, RNA extraction, flow cytometry analysis, and confocal microscopic analysis. After culture for 2 h at 37 °C, floating cells were removed, and the attached macrophages were further cultured for 24 h prior to use.

Peritoneal macrophages were treated with Alexa Fluor 488-labeled *S. aureus* Wood strain (Invitrogen, CA, USA) (multiplicity of infection (MOI) of 1 or 5) at 37 °C for 60 min for flow cytometry analysis; were treated with heat-killed *S. aureus* (MOI of 5) at 37 °C and then used for ELISA assays, RNA extraction, and confocal microscopic analysis at the indicated time points after treatment; or were untreated.

### Flow cytometry analysis of phagocytosis

The fluorescence of the extracellular bacteria adhered to the macrophage surface was quenched using 0.2% trypan blue, and cells were detached from dishes using a scraper and then resuspended in PBS. Cell-associated fluorescence was measured on a BD FACSAria and data were analyzed with a Diva software (both from BD Biosciences, CA, USA). For each sample, 10,000 events were analyzed. The results were expressed as the percentage of fluorescence-positive cells and fluorescence intensity that reflects the extent of macrophage phagocytosis of the bacteria.

### Confocal microscopic analysis

Peritoneal macrophages were incubated with rabbit anti-human CD36 (Santa Cruz Biotechnology Inc., CA, USA), followed by Alexa Fluor 488-conjugated chicken anti-rabbit IgG Ab (Molecular Probes, CA, USA). Immunostained cells were evaluated using laser-scanning confocal microscopy. Normal rabbit IgG (Santa Cruz Biotechnology Inc.) was used as a negative control.

### Quantification of cytokine production

ELISAs were used to determine cytokine concentrations from organ extracts and cell culture supernatants. The TNF-α level was determined as described previously [21]. A purified hamster anti-TNF-α mAb and a purified rabbit anti-TNF-α polyclonal Ab (Endogen, MA, USA) were used as the capture and detection Abs, respectively. A standard curve was constructed for each experiment by serially diluting recombinant TNF-α (R&D systems, MN, USA). IL-6 concentration was also measured by ELISA. Plates were coated with purified rat anti-IL-6 mAb (BD Bioscience, Inc.) and incubated with culture supernatants or organ extracts. IL-6 was detected with a biotinylated rat anti-IL-6 mAb (BD Bioscience, Inc.). All ELISAs were run with rIL-6 (BD Bioscience, Inc.). IL-10 concentrations were measured using the DuoSet ELISA development kit (R&D systems) according to the manufacturer’s protocol. The sensitivities of the ELISAs were 50 pg/ml for TNF-α and 20 pg/ml for IL-6 and IL-10.

### Quantitative real-time PCR analysis

Total RNA was isolated from peritoneal macrophages or mouse liver tissues using Trizol reagent (Invitrogen) and reverse transcribed to first-strand cDNA using the High-capacity cDNA reverse transcription kits (Applied Biosystems, CA, USA). The cDNA was utilized for quantitative real-time PCR with the Power 2 × SYBR Green PCR master mix and monitored on an ABI Prism 7000 sequence detection system (both from Applied Biosystems). The PCR conditions were as follows: 95°C for 10 min, followed by 40 cycles at 95 °C for 15 s and 60 °C for 1 min. The primer sequences of the selected genes are shown in Table 1. Relative expression of the target gene compared to that for the untreated control samples was normalized to the expression of the housekeeping gene GAPDH.

**Table 1:** Primers used for quantitative real-time PCR.

**Gene name**	**Direction**	**Primer sequence**	**Amplicon size (bp**)	**Accession No.**
TNF-α	Forward	5’-GGGTGATCGGTCCCCAAAGG-3’	95	NM_013693
	Reverse	5’-TTGAGAAGATGATCTGAGTGTGAGG-3’		
IL-6	Forward	5’-AAGAAATGATGGATGCTACCAAACTG-3’	79	NM_031168
	Reverse	5’-GTACTCCAGAAGACCAGAGGAAATT-3’		
IL-10	Forward	5’-GGACAACATACTGCTAACCGACTC-3’	73	NM_010548
	Reverse	5’-TTCCGATAAGGCTTGGCAACC-3’		
CD36	Forward	5’-TCTTATGGTGTGCTAGACATTGGC-3’	99	NM_007643
	Reverse	5’-AGGTTCTGAAACATCTGGACTTGC-3’		

### Histopathological examination

Animals were sacrificed, and liver samples were removed and fixed in 10% neutral formalin solution and then embedded in paraffin blocks for Gram-staining and immunostaining for F4/80 (a surface marker of mouse macrophages), CD36, and myeloperoxidase. To detect F4/80 and CD36, 2-µm-thick tissue sections were immunostained with rabbit anti-mouse F4/80 Ab (Acris Antibodies Inc., CA, USA) and rabbit anti-human CD36 Ab (LifeSpan Biosciences Inc., WA, USA), respectively, followed by detection using the STAT-Q rapid IHC system (Innovex Biosciences Inc., CA, USA). To detect myeloperoxidase, sections were stained with rabbit anti-human myeloperoxidase (Dako, Glostrup, Denmark), followed by detection using the Dako Envision Flex amplification kit (Dako). As a negative control, slides were incubated with normal rabbit IgG (Santa Cruz Biotechnology Inc.). Positive cells and stained regions were quantified using a Histo Quest software (TissueGnostics GmbH, Vienna, Austria), and the data are expressed as a percentage of the number of cells or a percentage of the field area.

### Statistical analysis

All the data are expressed as mean ± SD values. Significant differences between the values in the mouse groups were calculated using the Student’s *t* tests. *P*-values of < 0.05 were considered statistically significant.

## Results

### Survival of infected mice

**Figure 1:**
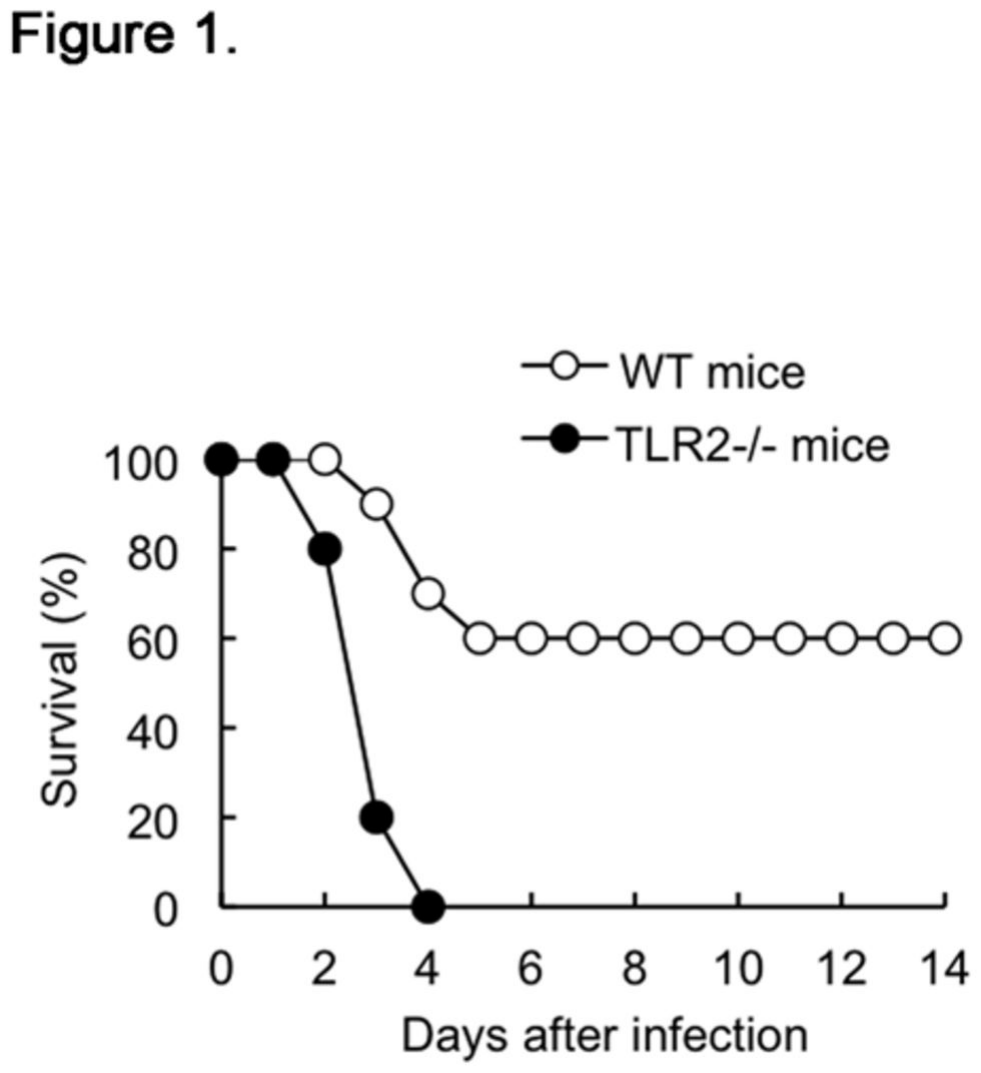
Survival rates of infected mice. After intravenous inoculation with 3 × 10^7^ CFU of *S. aureus*, the survival rates of WT mice and TLR2-deficient mice were determined over a 14-d experimental period. Survival curves were generated from 2 independent experiments, with a total of 20 mice per group.

After intravenous inoculation with 3 × 10^7^ CFU of viable *S. aureus*, the survival of WT and TLR2-deficient mice was monitored for up to 14 d. The TLR2-deficient mice were hypersusceptible to *S. aureus* infection and displayed high mortality rates compared to their WT counterparts. As shown in Figure 1, the WT mice exhibited a 60% survival rate at 5 d post-infection; thereafter, they survived the infection until 14 d post-infection. In contrast, TLR2-deficient mouse death began early at 2 d post-infection, and all succumbed to infection within 4 d post-infection (Figure 1). PBS injection had no effect on the survival of mice (data not shown).

### Bacterial loads and elimination in mouse organs

**Figure 2:**
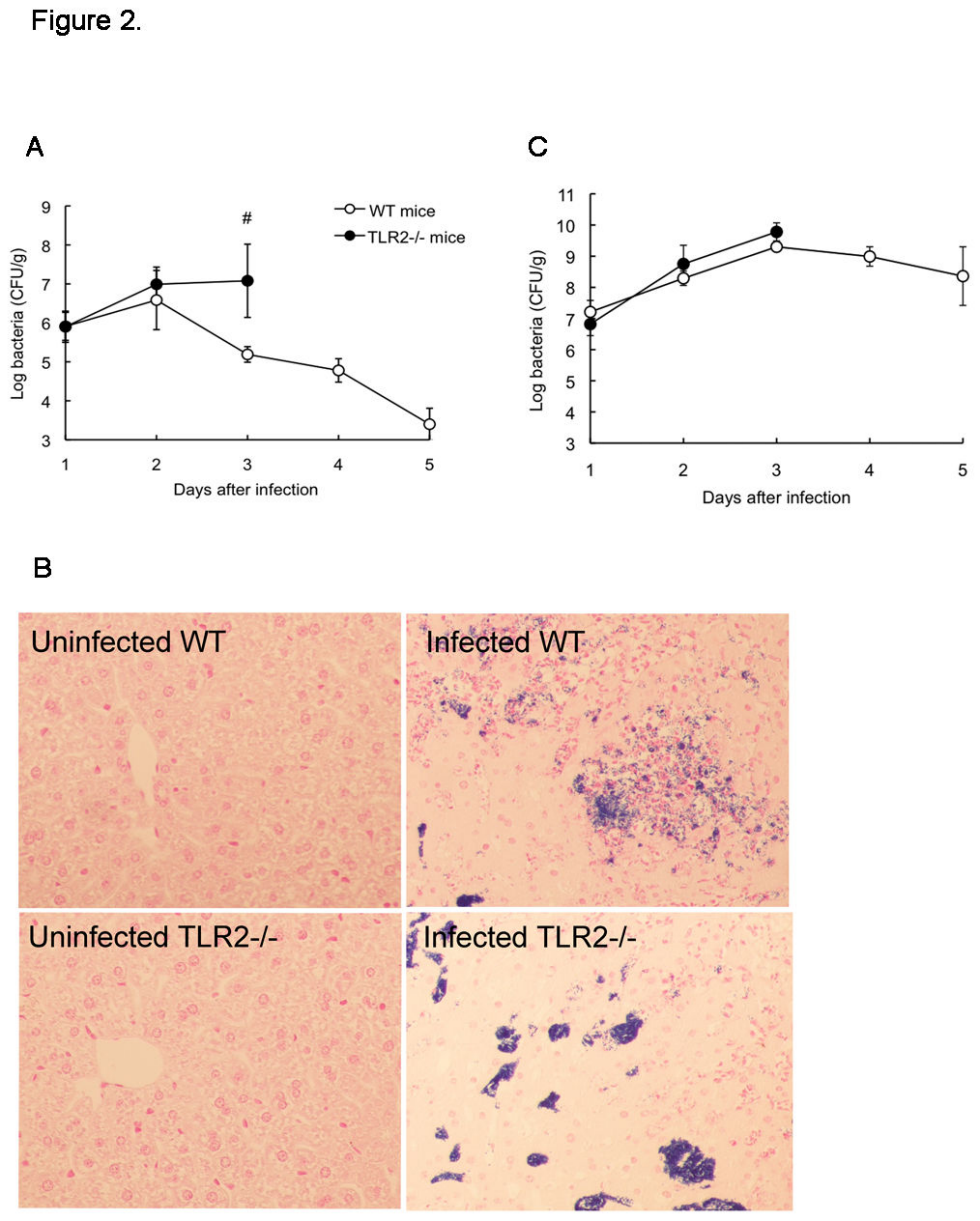
Kinetics of the bacterial load in murine organs. After infection with *S. aureus*, the counts of viable bacteria present in the livers (A) and kidneys (C) of WT and TLR2-deficient mice were determined from 1 to 5 d. Each point represents the mean ± SD of the *S. aureus* CFU from 2 independent experiments, with a total of 20–30 mice per group. ^#^ *p* < 0.01 vs. infected WT mice. (B) Representative Gram-stained photomicrographs (original magnification, × 100) of livers from uninfected mice and infected mice at 3 d post-infection are shown.

To determine whether the high mortality rates observed in TLR2-deficient mice were associated with an impaired capacity to control and eliminate *S. aureus*, we compared the *in vivo* kinetics of bacterial loads in the livers (a key organ of the innate immune system) and kidneys (a major target organ of *S. aureus*) of these mice to those in WT animals. A similar increase in the bacterial count was observed in the livers of WT and TLR2-deficient mice for up to 2 d post-infection; thereafter, the hepatic bacterial counts significantly reduced in the former, while the high levels of bacterial burden in the livers of the latter persisted until their death (Figure 2A). Similarly, Gram-stained liver sections of mice at 3 d post-infection revealed that a large number of *S. aureus* were present in clusters in the TLR2-deficient mice, while the bacteria were dispersed at infectious foci with few clusters in WT mice (Figure 2B). WT and TLR2-deficient mice also showed a same slight increase in bacterial count within 3 d post-infection in kidneys; thereafter, the WT mice exhibited reduced bacterial counts in the kidneys (Figure 2C).

### Phagocytic activity of macrophages and CD36 expression

**Figure 3:**
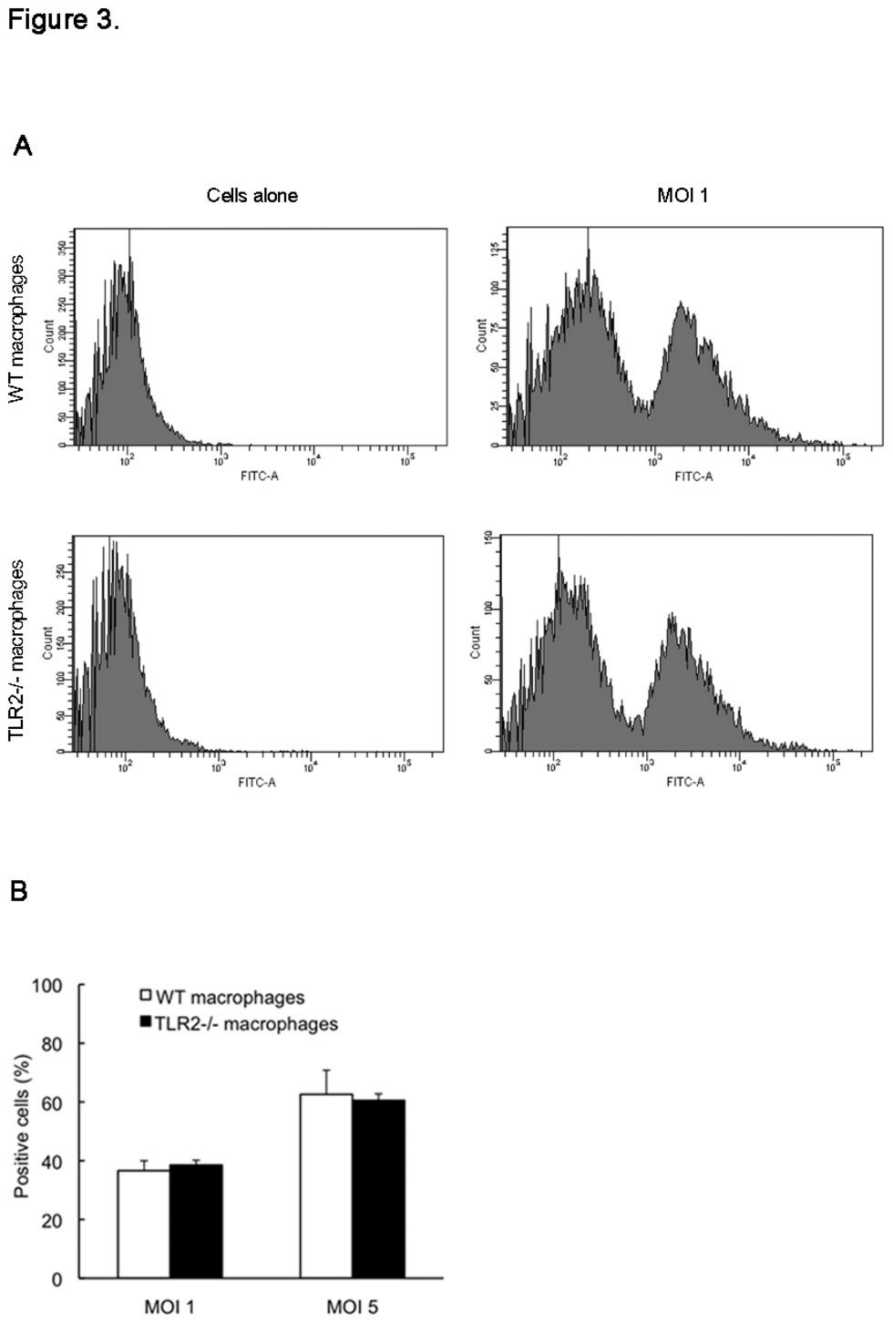
Phagocytosis of *S. aureus* by mouse macrophages. The peritoneal macrophages from WT and TLR2-deficient mice were left untreated or were treated for 60 min with Alexa Fluor 488-labeled *S. aureus* (MOI of 1 or 5) at 37°C and then harvested for flow cytometry analysis. (A) Histograms represent fluorescence intensity counts of 10,000 cells for each sample, and representative histograms are shown. (B) The percentage of positive cells that had engulfed bacteria is indicated. Data are expressed as the mean ± SD for 3 separate experiments.

**Figure 4:**
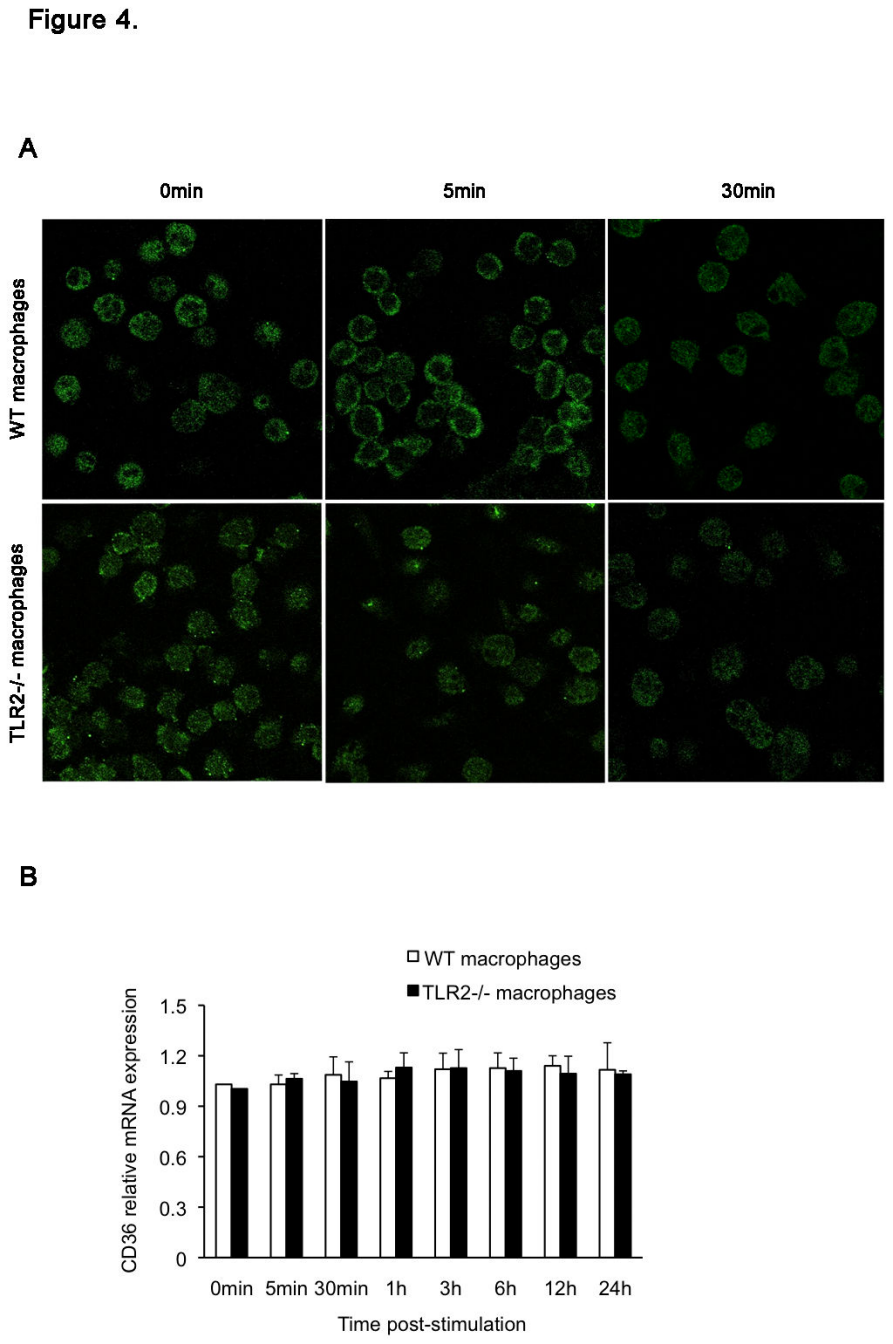
CD36 expression in mouse macrophages. Following treatment with heat-killed *S. aureus* (MOI of 5), the macrophages from WT and TLR2-deficient mice were examined for CD36 mRNA and protein expression by real-time PCR and laser-scanning confocal microscopy. Untreated mouse macrophages served as a control. (A) CD36 was present in the plasma of macrophages from both WT and TLR2-deficient mice under the no-treatment condition. Confocal microscopy showed rapid CD36 localization on the plasma membrane of the macrophages from WT mice at 5 min post-treatment; this expression rapidly disappeared thereafter. No change was observed in the cells from TLR2-deficient mice (original magnification, × 400). (B) The time course of CD36 mRNA expression in the 2 macrophage groups is shown. Results were normalized to GAPDH gene expression and are shown as fold changes relative to gene expression in the control cells. Data are expressed as the mean ± SD for 3 separate experiments.

We used AlexaFluor 488-labeled *S. aureus* to further investigate whether TLR2 deficiency alters the phagocytic activity of macrophages. As shown in Figure 3A, no significant difference was found in the degree of phagocytic engulfment of *S. aureus* between the macrophages of WT and TLR2-deficient mice at 60 min post-treatment. Moreover, the number of positive cells that engulfed the bacteria was the same for both macrophage groups (Figure 3B). Since the scavenger receptor CD36 has been reported to be involved in the macrophage uptake of *S. aureus* in cooperation with TLR2 [18], we next examined whether the absence of TLR2 affects CD36 expression in macrophages. Confocal microscopy showed that CD36 was present in the cytoplasm of macrophages from either WT mice or TLR2-deficient mice under the no-treatment condition (Figure 4A). Following treatment with heat-killed *S. aureus*, CD36 protein was observed to translocate onto the plasma membrane of the WT mouse macrophages as early as 5 min post-treatment and rapidly returned to the plasma subsequently (Figure 4A). In contrast, the treatment failed to alter CD36 protein expression in the macrophages from TLR2-deficient mice and no CD36 localization was observed on the plasma membrane (Figure 4A). In addition, there was no change in CD36 gene expression in both cell groups after exposure to heat-killed *S. aureus* (Figure 4B). These findings suggest that TLR2 and CD36 do not have a direct effect on macrophage phagocytosis of *S. aureus*. Moreover, CD36 activation in macrophages is dependent on the presence of the TLR2 molecule.

### *In vitro* kinetics of macrophage cytokine response

**Figure 5:**
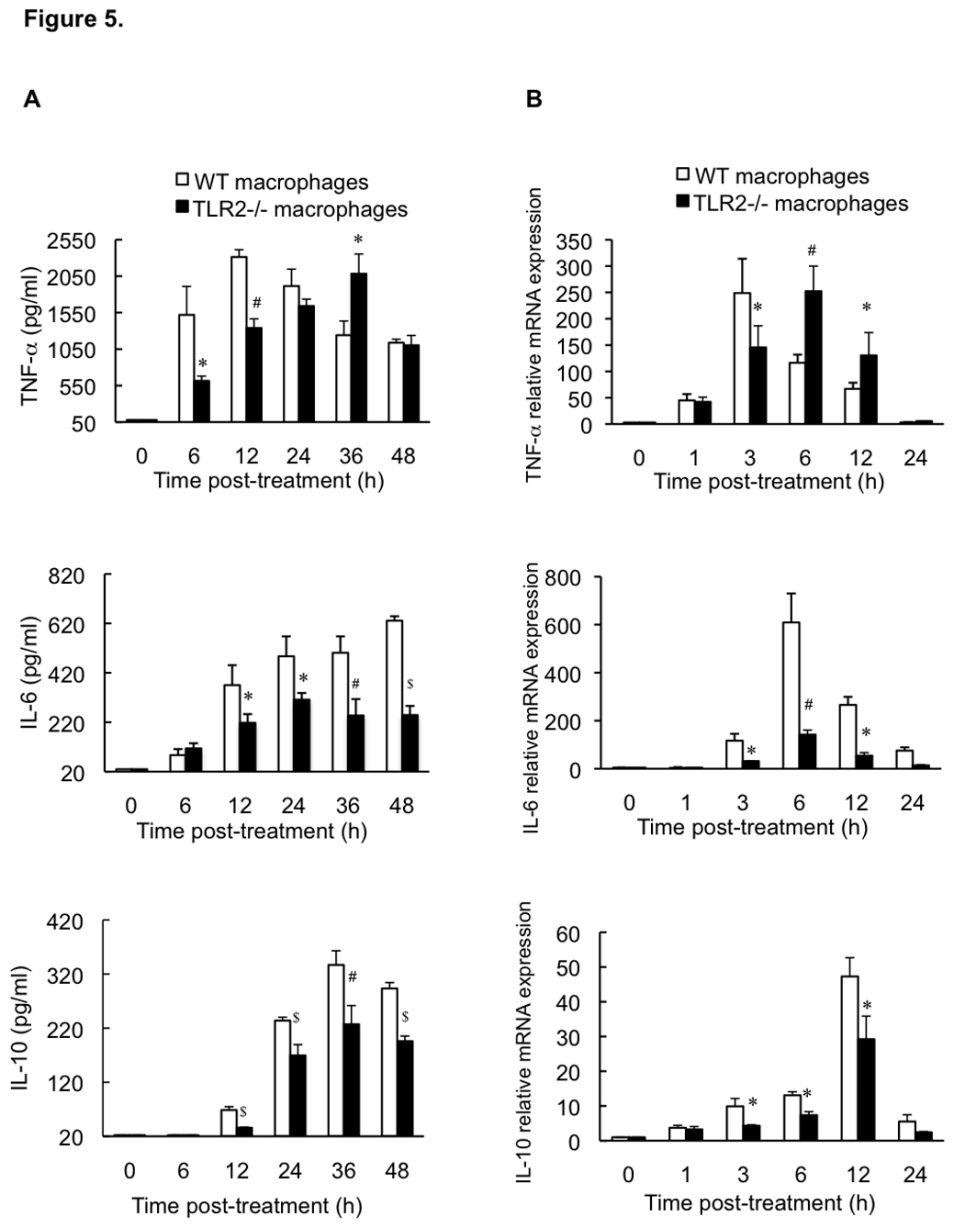
Kinetics of cytokine production (A) and mRNA expression (B) in mouse macrophages. Macrophages from WT mice and TLR2-deficient mice were untreated or treated with heat-killed *S. aureus* (MOI of 5), and then culture supernatants were harvested at different time intervals and assayed for TNF-α, IL-6 and IL-10 concentrations using ELISAs. Total RNA in these cells was also isolated and analyzed using real-time PCR for the mRNA expression of the cytokines at different time points. The results were normalized to GAPDH gene expression and are shown as fold changes relative to gene expression in untreated control cells. Data are the mean ± SD from 3 independent experiments. ^*^ *p* < 0.05, ^#^ *p* < 0.01, ^$^ *p* < 0.001 vs. WT mouse macrophages.

To ascertain the importance of TLR2 in the cytokine response to *S. aureus* infection in macrophages, we compared cytokine secretion by peritoneal macrophages from TLR2-deficient mice with those from WT mice. Macrophages from the 2 mouse groups did not produce cytokines under the no-treatment condition (Figure 5A). After exposure to heat-killed *S. aureus*, TLR2-deficient mouse cells exhibited delayed TNF-α production compared to that seen in WT mouse cells, although the peak levels observed in the former at 36 h were similar to those in the latter at 12 h (Figure 5A), suggesting that an alternative microbial recognition receptor might be involved. The macrophages from the WT mice also exhibited a lasting increase in IL-6 secretion in a time-dependent manner over a 48-h experimental period; in contrast, lower IL-6 secretion was detected in the cells from the TLR2-deficient mice, which peaked at 24 h post-treatment (Figure 5A). At 48 h post-treatment, a 61% decrease was seen in the IL-6 level in the cell culture supernatants from the TLR2-deficient mice relative to those from the WT mice (Figure 5A). Although treatment with heat-killed *S. aureus* resulted in similar IL-10 secretion kinetics in macrophages from WT mice and TLR2-deficient mice, IL-10 levels were markedly impaired in the latter, which showed a 32% reduction of IL-10 peak levels at 36 h post-treatment (vs. WT mouse cells; Figure 5A).

Cytokine production is regulated at both the transcriptional and translational levels. To determine whether the production of cytokines is associated with their mRNA expression, we evaluated cytokine mRNA expression using real-time PCR. As shown in Figure 5B, heat-killed *S. aureus* enhanced TNF-α, IL-6, and IL-10 mRNA levels in the macrophages from WT mice, which peaked at 3, 6, and 12 h post-treatment, respectively. In contrast, TLR2-deficient mouse macrophages showed significantly lower levels of IL-6 and IL-10 mRNA expression following treatment with heat-killed *S. aureus* and the peak levels of IL-6 and IL-10 decreased by 77% and 38% at 6 and 12 h post-treatment, respectively (vs. WT mouse cells; Figure 5B). Although the peak expression of TNF-α mRNA in TLR2-deficient mouse cells was similar to that in WT mouse cells, it was delayed by approximately 3 h (vs. WT mouse cells; Figure 5B). These gene expression patterns were also correlated with the levels of protein secretion. Treatment with heat-killed *S. aureus* did not affect GAPDH mRNA levels in macrophages. These results indicate that TLR2 deficiency results in a decreased macrophage cytokine response to heat-killed *S. aureus*.

### *In vivo* cytokine response in mice

**Figure 6:**
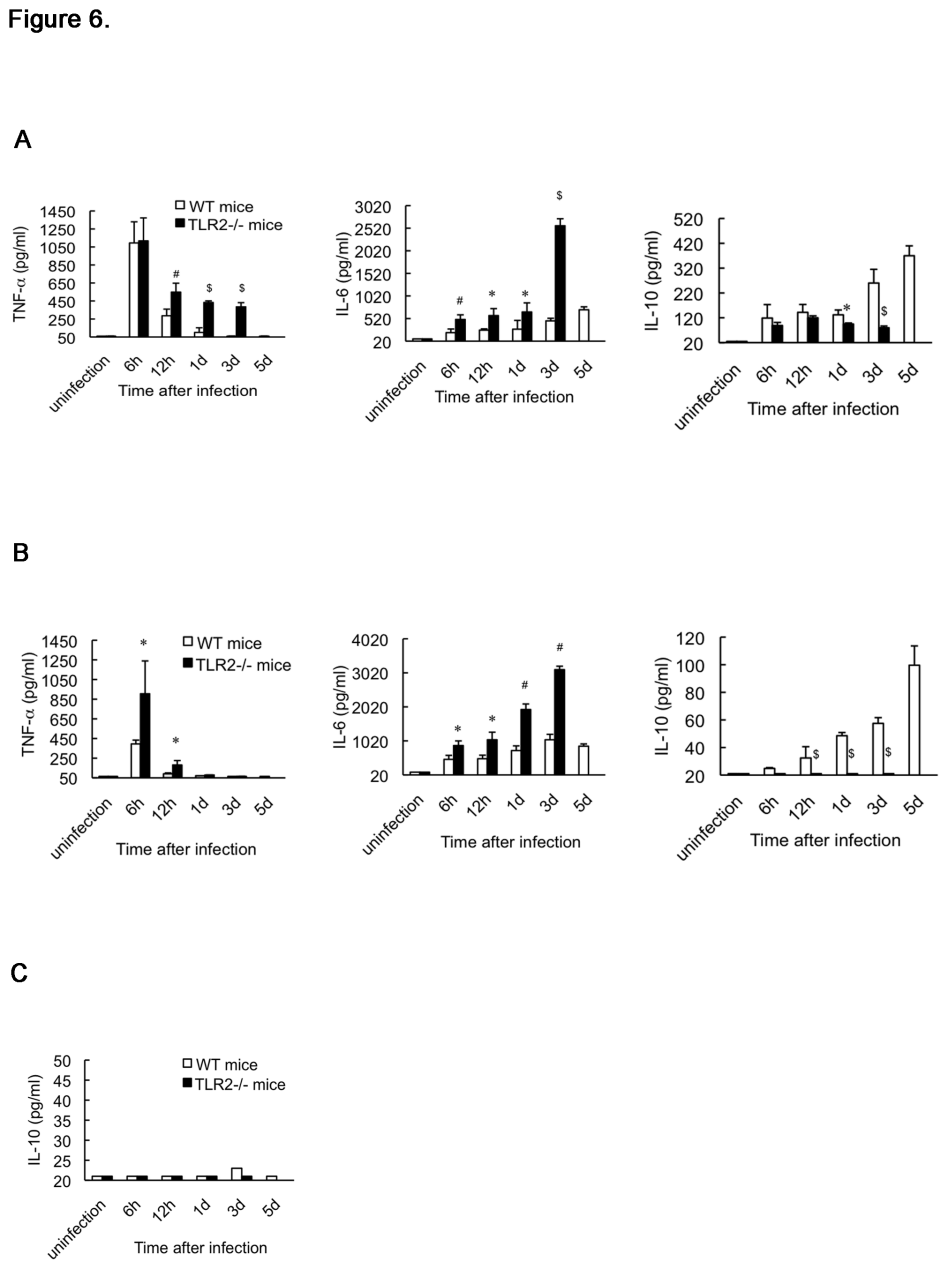
Cytokine production in the livers (A), blood (B), and kidneys (C) of mice. After inoculation with viable *S. aureus* or PBS, the concentrations of TNF-α, IL-6, and IL-10 in organ extracts and plasma from WT mice and TLR2-deficient mice at the indicated time intervals were assayed by ELISAs. Data are the mean ± SD from 3 independent experiments, with a total of 20–30 mice per infected group and a total of 6 mice per uninfected group. ^*^ *p* < 0.05, ^#^ *p* < 0.01, ^$^ *p* < 0.001 vs. infected WT mice.

An imbalance of pro- and anti-inflammatory cytokines generally results in an exaggerated inflammatory response, multiple organ dysfunction syndrome, and septic shock. Therefore, we examined the *in vivo* role of TLR2 in the cytokine response to *S. aureus* infection in mice. There was no cytokine production detected in the livers and blood of either uninfected (PBS-treated) WT or TLR2-deficient mice (Figure 6A, 6B). Inoculation with viable *S. aureus* in WT mice induced rapid elevation of TNF-α in the livers and blood at 6 h post-infection; thereafter, the TNF-α levels gradually diminished and were barely detectable at 3 d post-infection in livers and at 1 d post-infection in blood (Figure 6A, 6B). Although TLR2-deficient mice exhibited hepatic levels of TNF-α equivalent to those in WT mice at 6 h post-infection, a subsequent decrease in hepatic TNF-α levels was significantly delayed in TLR2-deficient mice with 7.7-fold higher levels at 3 d post-infection (vs. WT mice; Figure 6A). Moreover, blood TNF-α was also abundantly produced in the TLR2-deficient mice and peaked to levels 2.3-fold higher than those in WT mice at 6 h post-infection (Figure 6B). When WT mice were infected with *S. aureus*, IL-6 release was detected in the livers at 6 h post-infection and continued to gradually increase for at least until 5 d post-infection; it also appeared in the blood and peaked at 3 d post-infection (Figure 6A, 6B). In contrast, greater IL-6 elevation was seen in the organs of infected TLR2-deficient animals during their survival period; at 3 d post-infection, the hepatic and blood IL-6 levels were 5.5- and 2.9-fold higher than those in infected WT mice, respectively (Figure 6A, 6B). *S. aureus*-induced IL-10 production was also found in the livers and blood of WT mice at 6 h post-infection, and continued to increase through 5 d post-infection (Figure 6A, 6B). However, IL-10 release attenuated in the livers of TLR2-deficient mice from 1 d post-infection and significantly reduced by 70% at 3 d post-infection (vs. WT mice; Figure 6A). Furthermore, IL-10 levels were hardly detected in the blood of TLR2-deficient animals during infection (Figure 6B). These data suggest that TLR2 deficiency results in an increased pro-inflammatory response and attenuated anti-inflammatory response during systemic *S. aureus* infection. In addition, we also analyzed IL-10 production in murine kidneys. IL-10 release was not found in the kidneys of either WT mice or TLR2-deficient mice during infection (Figure 6C).

### Hepatic histopathology of mice

**Figure 7:**
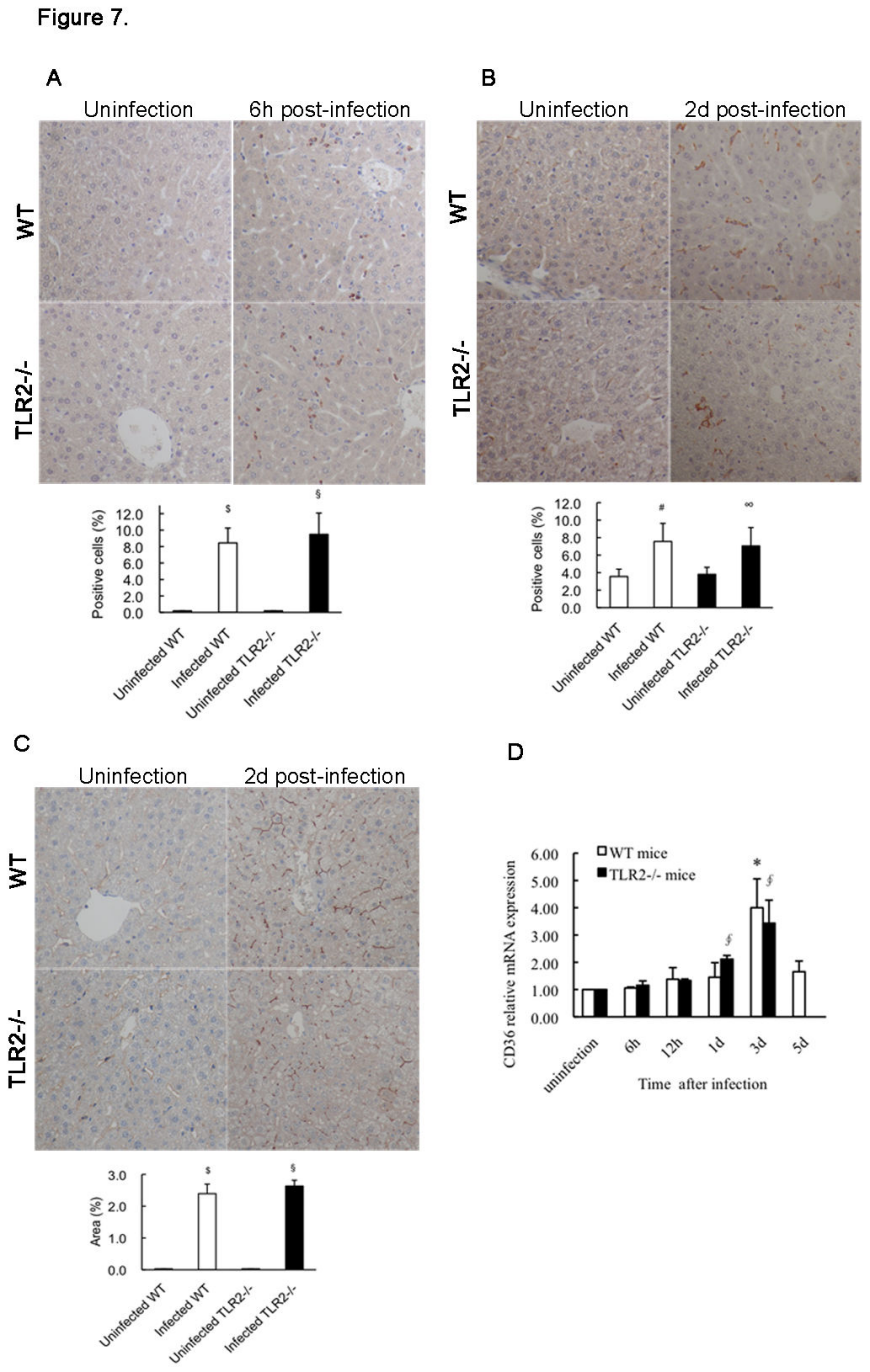
Inflammatory infiltration and CD36 expression in mouse livers. To identify the infiltrating cell types and CD36 expression in the livers of infected WT mice and TLR2-deficient mice, murine liver tissues were immunostained for neutrophils, macrophages, and CD36, and analyzed for the CD36 gene expression level by real-time PCR. Representative photomicrographs (original magnification, × 200) of liver sections stained with an anti-MOP antibody for neutrophils at 6 h post-infection (A); an F4/80 antibody for macrophages at 2 d post-infection (B); and an anti-CD36 antibody for CD36 protein expression at 2 d post-infection (C); and the percentage of positively stained cells and the percentage of positively stained field areas are shown. Uninfected WT mice and TLR2-deficient mice served as controls. (D) Results from real-time PCR were normalized to GAPDH gene expression and are shown as fold changes relative to gene expression in the control mice. Data are presented as the mean ± SD for 5–7 mice per infected group and for 3 mice per uninfected group. ^*^ *p* < 0.05, ^#^ *p* < 0.01, ^$^ *p* < 0.001 vs. uninfected WT mice; ^∮^ *p* < 0.05, ^∞^ *p* < 0.01, ^§^ *p* < 0.001 vs. uninfected TLR2-deficient mice.

We compared the hepatic inflammatory infiltration in WT and TLR2-deficient mice by histological examination. There were a few Kupffer cells (hepatic resident macrophage population) in the livers of untreated WT and TLR2-deficient mice (Figure 7B). Inoculation with *S. aureus* resulted in rapid neutrophil infiltration into the hepatic sinusoid of both the WT mice and TLR2-deficient mice within the first 6 h post-infection; at 2 d post-infection, an increase in macrophages was noted in the livers of the 2 mouse groups (Figure 7A, 7B). However, no statistically significant difference was observed between the 2 groups with respect to inflammatory infiltration (Figure 7A, 7B). These data suggest that the *S. aureus*-induced inflammatory cell accumulation is not dependent on the presence of TLR2.

It has been reported that CD36 expression is normally low in the rodent liver [22]. Similarly, no CD36 expression was found in the livers of uninfected WT and TLR2-deficient mice (Figure 7C). After *S. aureus* infection, CD36 expression gradually increased on the surface of the hepatocytes in either WT mice or TLR2-deficient mice and was obvious at 2 d post-infection; however, there was no difference in CD36 expression between the 2 mouse groups (Figure 7C). Notably, this observation was also supported by mRNA levels (Figure 7D).

## Discussion

In the present study, we analyzed the contribution of TLR2 to both bacterial clearance and cytokine response to *S. aureus* infection in mice. Several studies have shown increased mortality in TLR2-deficient mice compared to that in WT mice during *S. aureus* infection, which is thought to be due to TLR2 deficiency-induced defective phagocytosis, high bacterial tissue burden, and the impairment of pro-inflammatory cytokine production [12,17,18]. Similarly, in this study, TLR2-deficient mice showed enhanced susceptibility and all succumbed to *S. aureus* infection within 4 d post-infection as well as a high bacterial burden in their livers. However, contrary to previous reports, an excessive pro-inflammatory cytokine response was observed in these mice. In terms of *S. aureus* clearance, professional phagocytes such as macrophages and neutrophils, constitute the first line of host innate immunity to engulf and kill the bacterium [8,23]. Although the TLR2 on phagocytes does not function as a phagocytic receptor, its absence is reported to cause a decrease in bacterial phagocytosis [24,25]. However, scattered evidence suggests that *S. aureus* is phagocytosed by macrophages in a TLR2-independent manner and that TLR2-deficient mice exhibit phagocytic and bactericidal functions in neutrophils similar to those seen in WT mice during *S. aureus* infection [26,27]. These divergent data prompted us to re-examine the role of TLR2 in *S. aureus* clearance.

The liver is an important immunologic organ that takes up and eliminates most bacteria that enter the bloodstream. During the first 2 d post-infection, WT mice and TLR2-deficient mice displayed the same levels of bacterial load in livers as well as equal accumulation of neutrophils and macrophages. Moreover, the results from flow cytometry analysis showed that TLR2 deficiency did not alter phagocytic uptake of *S. aureus* by macrophages. On the basis of these observations, we think that TLR2 does not contribute to the early clearance of *S. aureus*. In addition, our findings also support the view that accumulation of leukocytes including the early neutrophils and the later macrophages, at the infectious site is unaffected by TLR2 deficiency [27,28]. The class B scavenger receptor CD36 has been reported to predominantly mediate *S. aureus* clearance by murine phagocytes, and CD36-deficient mice fail to efficiently remove *S. aureus in vivo* resulting in profound bacteremia and high mortality [12,18,29]. In this study, confocal microscopic analysis showed a significant difference in macrophage CD36 expression between the WT and TLR2-deficient mice in response to heat-killed *S. aureus*. Immediately after treatment, rapid and transient expression of CD36 was observed on the plasma membrane of WT mouse macrophages, but this did not occur on TLR2-deficient mouse cells. This treatment did not alter CD36 gene expression in either cell group. Our results indicate that the transient translocation of CD36 protein does not have an effector function in macrophage phagocytosis of *S. aureus*. Similar to our studies, work performed by Hawkes et al. also showed that CD36 is not required for mycobacterial phagocytosis and clearance by macrophages [30]. In addition, the *S. aureus*-induced rapid modulation of CD36 is considered to be due to pre-synthesized protein in the cells but is not related to new protein synthesis. With regard to the interaction between CD36 and TLR2 on macrophages, CD36 has been found to initiate the TLR2 signaling pathway when it binds and engulfs particles including *S. aureus* and its PAMPs [18,31,32]. However, there are few studies demonstrating the effect of TLR2 on CD36 expression upon bacterial infection. To the best of our knowledge, this is the first report demonstrating that the CD36 expression on macrophages in response to *S. aureus* infection depends on the presence of TLR2. In addition, it is notable that CD36 protein clearly appeared on the surface of hepatocytes in both WT and TLR2-deficient mice at 2 d post-infection along with a corresponding increase in gene expression, suggesting that TLR2 is not required for this process. Hence, we conclude that CD36 expression on different cells is regulated by distinct mechanisms. However, the regulatory mechanisms and the function of CD36 during *S. aureus* infection remain unclear and require further investigation.

Previous studies indicated that *S. aureus*-induced activation of TLR2 leads to the release of pro-inflammatory cytokines by immune and non-immune cells, especially by macrophages, in which TNF-α is considered to be an early cytokine while IL-6 is considered to be a later cytokine [15,33,34]. However, the anti-inflammatory IL-10 response has also been identified to occur in macrophages/monocytes upon TLR2 signaling initiated by *S. aureus* [35,36,37]. In our *in vitro* experiments, macrophages from TLR2-deficient mice showed impaired, but not completely depressed, release of IL-6 and IL-10 in response to heat-killed *S. aureus*. Moreover, these cells also exhibited delayed TNF-α production compared to that in macrophages from WT mice. These observations indicate that the macrophage response to *S. aureus* infection, including both pro- and anti-inflammatory responses, does not entirely depends on TLR2 signaling. Several studies have also demonstrated that not only TLR2, but also other microbial recognition receptors, such as intracellular nucleotide-binding oligomerization domain-like receptors, are involved in evoking cytokine responses to *S. aureus* infection [5,38].

We further investigated the *in vivo* cytokine responses triggered upon viable *S. aureus* infection. Although the 2 mouse groups displayed the same degree of hepatic macrophage and neutrophil accumulation after inoculation with the bacteria, a hyper-elevated pro-inflammatory cytokine response was clearly observed in the livers and blood of TLR2-deficient mice relative to that in the WT mice. Our *in vitro* experiments showed that TLR2 deficiency attenuated macrophage secretion of pro-inflammatory cytokines during *S. aureus* infection. Thus, the pro-inflammatory cytokine response was predominantly mediated by rapidly recruited neutrophils. Recent studies have identified that the neutrophil is a major source of pro-inflammatory cytokines and that exposure of neutrophils to *S. aureus* results in TNF-α and IL-1β release by a TLR2-independent mechanism [27,39]. Moreover, IL-1β exhibits the capacity to strongly amplify the nucleotide-binding oligomerization domain 2-dependent IL-6 response to *S. aureus* [40]. Besides releasing pro-inflammatory cytokines, neutrophils also generate chemotactic factors that activate other cells to participate in the inflammatory response to bacterial infection [41,42]. In this study, we also showed a markedly diminished IL-10 response in TLR2-deficient mice in response to *S. aureus* infection, consistent with *in vitro* results. IL-10 has been reported to ameliorate the outcome of *S. aureus* infection by promoting bacterial clearance, whereas IL-10 inhibition by neutralizing antibody or genetic deletion increases tissue bacteria burden [43,44]. Sasaki et al. also indicated the effect of IL-10 on host resistance to *S. aureus* infection [45]. Considering our observations and these previous reports, we think that the high hepatic bacterial load seen in TLR2-deficient mice at the late phase of infection (3 d post-infection) was due to a lasting decrease in IL-10 release. The findings that a large number of bacteria persisted in the kidneys of both WT and TLR2-deficient mice in which no IL-10 release was detected, further support our idea. Since the initial pro- and anti-inflammatory cytokine balance is most probably decisive for the total inflammatory outcome of a bacterial infection and the host survival [21,35,46,47], the excessive pro-inflammatory response to *S. aureus* infection observed in TLR2-deficient mice is thought to be due to markedly impaired IL-10 production, at least partly, and this unbalanced cytokine response ultimately results in mouse death. Additionally, it is also possible that the flourishing bacteria in the livers of TLR2-deficient mice at the late phase of infection further accelerate pro-inflammatory cytokine induction.

Taken together, in this study, we find the novel facts that TLR2 and scavenger receptor CD36 do not play a pivotal role in the direct clearance of *S. aureus* and that the CD36 activation in macrophages depends on the presence of TLR2. Our work also demonstrates that TLR2 deficiency causes reduction in IL-10 release by macrophages during *S. aureus* infection, which is thought to be responsible for dysregulated cytokine balance, impaired bacterial clearance, and mouse death. Therefore, TLR2 has a protective effect during systemic *S. aureus* infection due to its ability to control the balance of pro- and anti-inflammatory cytokines.
